# Sub-10 nm beam confinement by X-ray waveguides: design, fabrication and characterization of optical properties

**DOI:** 10.1107/S0909049511051983

**Published:** 2012-01-06

**Authors:** S. P. Krüger, H. Neubauer, M. Bartels, S. Kalbfleisch, K. Giewekemeyer, P. J. Wilbrandt, M. Sprung, T. Salditt

**Affiliations:** a Institut für Röntgenphysik, Universität Göttingen, Friedrich-Hund-Platz 1, 37077 Göttingen, Germany; b Institut für Materialphysik, Universität Göttingen, Friedrich-Hund-Platz 1, 37077 Göttingen, Germany; c Deutsches Elektronen-Synchrotron DESY, Notkestrasse 85, 22607 Hamburg, Germany

**Keywords:** X-ray waveguides, X-ray imaging

## Abstract

The propagation of hard X-ray synchrotron beams in waveguides with guiding layer diameters in the 9–35 nm thickness range has been studied. The planar waveguide structures consist of an optimized two-component cladding. The presented fabrication method is suitable for short and leak-proof waveguide slices with lengths (along the optical axis) in the sub-500 µm range, adapted for optimized transmission at photon energies of 11.5–18 keV. A detailed comparison between finite-difference simulations of waveguide optics and the experimental results is presented, concerning transmission, divergence of the waveguide exit beam, as well as the angular acceptance. In a second step, two crossed waveguides have been used to create a quasi-point source for propagation-based X-ray imaging at the new nano-focus endstation of the P10 coherence beamline at Petra III. By inverting the measured Fraunhofer diffraction pattern by an iterative error-reduction algorithm, a two-dimensional focus of 10 nm × 10 nm is obtained. Finally, holographic imaging of a lithographic test structure based on this optical system is demonstrated.

## Abstract 2

Optimized X-ray waveguides have been fabricated and characterized in terms of transmission, angular acceptance, farfield pattern and imaging applications. Beam confinement down to sub-10 nm in two orthogonal directions has been demonstrated, at the nano-focus endstation at P10 of PETRA III at HASYLAB/DESY.

## 1. Introduction

X-ray waveguides can be used for spatial and coherent filtering of X-rays (Lagomarsino *et al.*, 1997 ▶; Pfeiffer *et al.*, 2002 ▶; De Caro *et al.*, 2003 ▶; Jarre *et al.*, 2005 ▶; Osterhoff & Salditt, 2009 ▶). Using waveguides as a filter, it is possible to decouple the coherence of the exit beam from the primary source. Notably, the limit of full coherence is reached for a suitable choice of the guiding and the cladding material, as soon as the waveguide supports only a single mode (mono-modal propagation) below a (material-dependent) critical thickness 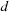 of the guiding layer. Along with other focusing optical devices (Hignette *et al.*, 2005 ▶; Schroer & Lengeler, 2005 ▶; Chao *et al.*, 2005 ▶; Kang *et al.*, 2008 ▶), waveguides serve to improve resolution in X-ray holography and coherent diffractive imaging (CDI) (Eisebitt *et al.*, 2004 ▶; Fuhse *et al.*, 2006 ▶; Quiney *et al.*, 2006 ▶; De Caro *et al.*, 2008 ▶; Thibault *et al.*, 2008 ▶). Similar to other reflective optical components, waveguides are essentially non-dispersive, and can be adapted to a wide range of photon energies and bandpass. The high spatial coherence and small beam cross section in the sub-20 nm range of X-ray waveguides has been utilized for propagation projection imaging of biological specimen in the hard X-ray range (Giewekemeyer *et al.*, 2011 ▶). The main remaining challenge in X-ray waveguide optics is to overcome fabrication difficulties and low transmission, *i.e.* absorption losses in the cladding.

We have previously shown that a two-component cladding design (Salditt *et al.*, 2008 ▶) can significantly enhance the transmission 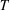 of the waveguide which is a prerequisite for high-resolution imaging. Furthermore, the transmission of the waveguide is intrinsically related to the waveguide length 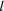. In this paper we study waveguide properties, and in particular transmission, of different waveguide lengths down to 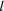 = 200 µm, and guiding layer thicknesses 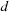 down to 9 nm cross section. Small cross sections are realised using magnetron sputtering of the optical films (C, Mo, Ge) whereas short waveguide lengths are enabled by novel cap wafer designs which efficiently block over-illumination and stray radiation. Planar one-dimensionally confining waveguides (1DWG) can be extended to two-dimensionally confining waveguides (2DWG) where two 1DWG slices with thicknesses in the range of a few hundred micrometres are glued onto each other in a crossed geometry (Krüger *et al.*, 2010 ▶).

Based on this crossed waveguide approach, and using the nano-focus endstation of the P10 coherence beamline at Petra III, we have achieved 10.0 nm and 9.8 nm beam confinement (full width at half-maximum, FWHM) in the respective horizontal and vertical focal planes, with an integrated photon flux of 2.0 × 10^7^ photons s^−1^, measured at 15 keV photon energy. For a second pair of waveguides cut to a smaller total length, an even higher flux of 1.0 × 10^8^ photons s^−1^ with a cross section of 10.7 nm and 11.4 nm FWHM in the horizontal and vertical direction, respectively, was measured at 13.8 keV photon energy, as detailed below. These results can be compared with two previous approaches reported in the literature for ultra-small X-ray beam generation: (i) elliptical multilayer mirrors with which the Osaka group has achieved the 7 nm record in hard X-ray focusing, as published for one-dimensional focusing by Mimura *et al.* (2010 ▶), and most recently extended to two-dimensional sub-10 nm focusing (unpublished); (ii) focusing by a crossed multilayer Laue lens (MLL), with a reported focus of 25 nm × 27 nm (Yan *et al.*, 2011 ▶). The best choice of the optical system for a particular imaging application depends on the experimental requirements. To mention some major differences, MLL and elliptical mirrors yield a beam focus which is freely accessible, while the effective focal plane of a waveguide is located at the device exit and is thus not freely accessible for a sample. Thus waveguide illumination is restricted to propagation imaging with the sample positioned in a defocus position downstream from the waveguide exit. On the other hand, the fact that any radiation outside the guiding layer is efficiently blocked in the cladding can be a clear advantage of waveguide nano-beams over MLL or mirror focusing which exhibit background radiation by zero and higher-focusing orders, or pronounced tail scattering, respectively. Finally, the coherence properties of the waveguide exit beam are decoupled from the source, and, after beam filtering by propagation within the waveguide, a fully coherent beam is easily achieved (Osterhoff & Salditt, 2011 ▶).

## 2. Waveguide design, theory and simulation

**Figure 1:**
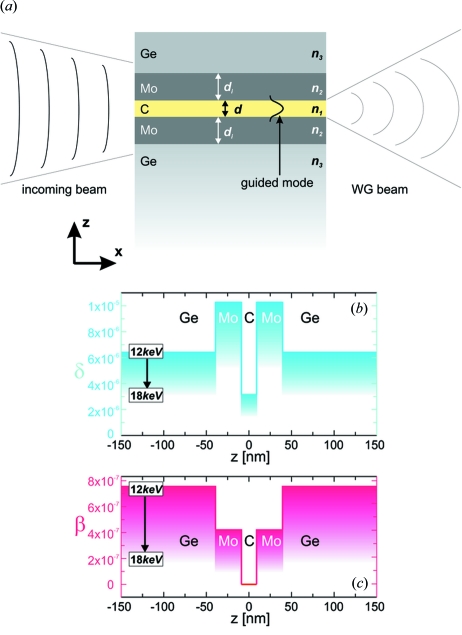
(*a*) Schematic of the Ge/Mo/C/Mo/Ge waveguide and the X-ray beam filtering. We have investigated different guiding layer thicknesses 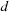 of the C layer in the experiments. Profiles of the real δ (*b*) and the imaginary β (*c*) part of the refractive index 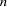 = 1 + 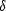 − 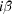 of the multilayer waveguide calculated for energies between 12.0 keV and 18.0 keV. The transmission efficiency of the two-component waveguide is enhanced owing to the relatively high 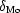 but low 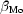 of the Mo interlayer.

The transmission efficiencies of planar waveguides are significantly enhanced by placing an appropriate interlayer between the strongly absorbing substrate and the guiding layer as introduced by Salditt *et al.* (2008 ▶). To this end, we chose a Ge/Mo/C/Mo/Ge optical layer sequence where the C guiding layer of the waveguide is embedded in Mo interlayers. The waveguide design along with the profiles of the refractive index are shown in Fig. 1 ▶. The waveguide is illuminated by an essentially unfocused beam on the front side (front-coupling waveguide), as shown in Fig. 1(*a*) ▶. Depending on the guiding layer thickness 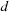, one or more modes are excited inside the waveguide, leading to a coherent (in the case of mono-modal propagation) quasi-spherical beam exiting the waveguide. The profiles (real and imaginary components) of the X-ray refractive index *n* = 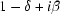 are visualized for a range of photon energies 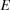 = 12–18 keV; see Figs. 1(*b*) and 1(*c*) ▶. The C layer embedded in the high-δ Mo cladding forms a relatively deep potential well. At the same time, a relatively low β value of Mo reduces the absorption in the (interlayer) cladding and hence enables an increased transmission 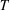. For example, at 12.0 keV the relatively low 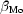 = 4.2 × 10^−7^ of the thin Mo interlayer compares with 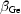 = 7.3 × 10^−7^ of the Ge capping layer. This design reduces the absorption of the propagating modes at the interfaces of the guiding layer. The higher the value of 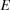, the more pronounced is this effect. In addition, the relatively high 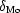 = 1.3 × 10^−5^–5.5 × 10^−6^ at 12.0–18.0 keV compared with 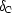 = 3.2 × 10^−6^–1.4 × 10^−6^ enables confinement of modes to smaller 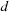 and larger angular acceptance, as compared with waveguides with a single-component cladding. The C layer absorption 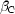 = 2.0 × 10^−9^–3.5 × 10^−10^ at 12.0–18.0 keV is two orders of magnitude lower than 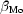, *i.e.* the contribution of the guiding layer to the effective absorption 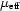 is less than 2%.

**Figure 2:**
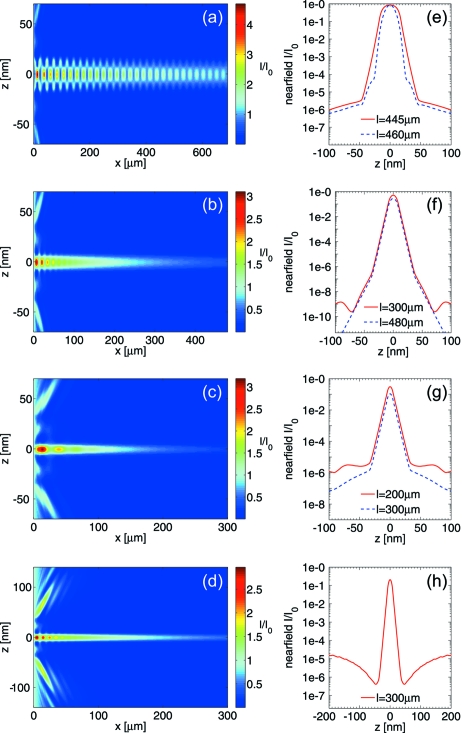
Simulated electromagnetic field intensities of the Ge/Mo/C/Mo/Ge waveguide at 15.0 keV [(*a*) 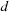 = 35 nm C layer, (*b*) 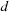 = 18 nm C layer, (*c*) 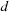 = 9 nm C layer] and of the Ge/Ni/C/Ni/Ge waveguide at 8.0 keV [(*d*) 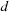 = 18 nm C layer]. The simulations show two-mode propagation in (*a*) and mono-modal propagation in (*b*)–(*c*). (*e*)–(*h*) Simulated near-field distributions in the exit plane corresponding to (*a*)–(*d*), respectively. The data show how the exit fields depend on the waveguide length and the guiding layer thickness.

Propagation of X-rays in planar waveguides can be described by the parabolic wave equation (Fuhse *et al.*, 2004 ▶; Panknin *et al.*, 2008 ▶). Taking into account attenuation of the electromagnetic field inside the waveguide by introducing an effective linear attenuation coefficient 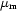, the solution of the parabolic wave equation sufficiently far away from the entrance reads
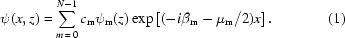
Hence, the wavefield inside the waveguide is given by a superposition of guided modes 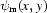. Here, 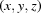 denotes the Cartesian coordinate system where 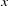 is the propagation direction. 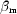 denotes the propagation constant of the different guided modes. The coefficients 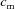 are given by the projection of the incident field 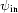 onto the respective eigen functions 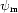,
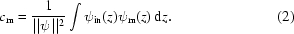
The attenuation coefficient 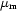 depends on the attenuation of the wavefield in the guiding layer 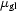 as well as in the interlayer 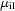 and is given by
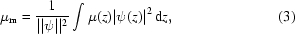
with
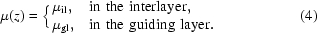
The wavefield of (1) is a solution of the parabolic wave equation when the characteristic equation is fulfilled (Fuhse, 2006 ▶). This equation leads to the waveguide parameter *V* ≃ 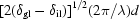, determining the number of modes propagating through the waveguide by *N* = 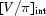, where 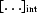 indicates the term is rounded up to the next integer. A planar waveguide supports only one guided mode if the guiding layer thickness 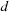 is smaller than a critical thickness *W* = 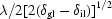. The critical thickness is 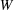 ≃ 12 nm for a Mo interlayer and a C guiding layer. X-ray propagation through waveguides has been studied using analytical theory as well as finite-difference (FD) simulations (De Caro *et al.*, 2003 ▶; Fuhse & Salditt, 2006 ▶; Bukreeva *et al.*, 2006 ▶, 2011 ▶) for more general waveguide designs including the two-component cladding waveguides. We chose a Crank–Nicolson-like finite-difference scheme to solve the parabolic wave equation and to simulate electromagnetic field distribution inside planar waveguides. We assumed an incident plane wave impinging onto the front side of the waveguide in numerical simulations. Figs. 2(*a*)–2(*c*) ▶ show the electromagnetic field distribution for varying guiding layer thicknesses 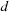 and different waveguide lengths 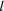 at 15.0 keV, using the code and similar simulation parameters as in Fuhse & Salditt (2006 ▶).

As expected, a waveguide (WG) with a 35 nm guiding layer supports three modes leading to a periodically alternating field distribution, as shown in Fig. 2(*a*) ▶. As the wavefield propagates through the waveguide, the third mode is damped out by absorption in the cladding. Depending on the exact length of the WG slice, different near-field profiles are obtained, as shown in Fig. 2(*e*) ▶. The FWHM of the near-field distribution varies between 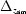 = 15.8 nm and 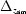 = 29.8 nm for WG lengths 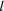 = 450 ± 15 m. It is very difficult to obtain direct information on the electromagnetic field inside a waveguide and on the near-field distribution at the exit side of the waveguide in an experiment. However, it is possible to deduce the information on the near-field distribution by inversion of the coherent far-field Fraunhofer diffraction pattern given by
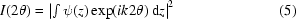
as a function of the exit angle 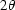, where 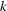 denotes the wavenumber. Thus, the measured far-field diffraction pattern is related to the exit-field distribution and may change according to the exact length of the WG slice. In the case of 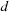 = 18 nm shown in Fig. 2(*b*) ▶, the absorption in the cladding damps out the second mode and, after a propagation length of 300 m, the fundamental mode is the only remaining mode.

Note that the simulated near-field width (FWHM) of the intensity 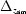 = 12.5 nm is smaller than the guiding layer, which confines the mode. For the 9 nm WG the intensity narrows down to 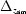 = 8.6 nm, which is significantly lower than that of the 18 nm WG, but only sightly lower than the guiding layer, owing to increasing intensity tails in the cladding. Two-component cladding waveguides with a Ge/Mo/C/Mo/Ge optical film sequence and with very short WG lengths are mainly suitable above the Ge absorption edge at 11.3 keV. For imaging experiments at a lower X-ray energy range, different material combinations can be chosen. For example, at 8.0 keV, a Ge/Ni/C/Ni/Ge-WG with 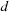 = 18 nm and 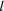 = 300 m leads to an optimized transmission and near-field width (FWHM) of 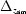 = 12.9 nm, as shown in Figs. 2(*d*) and 2(*h*) ▶.

## 3. Waveguide fabrication

**Figure 3:**
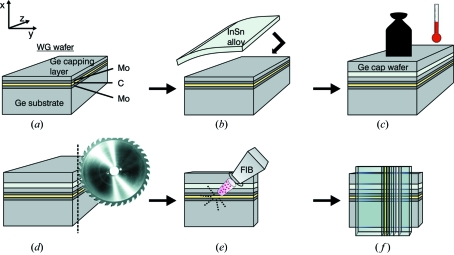
Schematic illustrating the different steps of the waveguide fabrication (see text).

**Figure 4:**
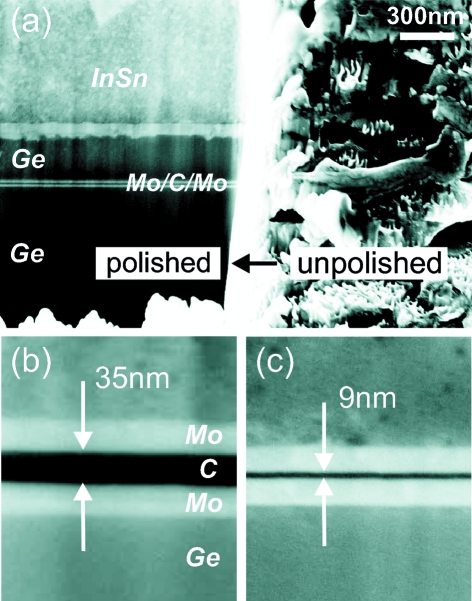
(*a*) The polished and unpolished exit side of the waveguide slices are polished, illustrating the beneficial effect of the focused ion beam technique in cleaning the optical layers. In the scanning electron microscopy images (*b*) and (*c*), the 35 nm C and 9 nm C guiding layers along with the interlayers are clearly identified (magnification 200k× and 300k×, respectively).

The different steps of waveguide fabrication are shown schematically in Fig. 3 ▶: (*a*) The thin-film structure is deposited by magnetron sputtering (Incoatec GmbH, Geesthacht, Germany), consisting of the C guiding layer in between two Mo interlayers on a 3 mm-thick Ge substrate [single-crystal (100) orientation] with low interface roughnesses (sub-5 Å). An approximately 1 m-thick Ge layer is sputtered onto the Mo/C/Mo multilayer which acts as a first capping layer. (*b*) Two techniques are used to enlarge the capping layer above the Ge layer, feasible to block synchrotron beams in the hard X-ray energy range. The first technique consists of bonding a cap wafer (Ge, 440 m thickness) onto the WG wafer by an alloying process. To ensure sufficient adhesion and wetting of the alloy, a 3 nm-thick Cr interlayer and a 120 nm-thick Ni interlayer was deposited both on the WG and cap wafer by electron beam evaporation. Bonding was achieved by an In52Sn48 alloy [GPS Technologies GmbH, indalloy number 1E (*T*_solidus_ = 391 K)] ‘sandwiched’ between the Ni faces of the WG and cap wafers, under a pressure of 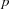 = 1 bar and heated up to 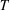 = 423 K under vacuum conditions (sub-1 mbar), as shown in subfigure (*c*). Next, (*d*) shows the dicing of the waveguide ‘sandwich’ into slices with waveguide lengths down to sub-150 m using a wafer dicer (dicing saw: DISCO DAD 321; diamond dicing blade: DISCO NBC-ZB 1070, 59 × 0.15 × 40; feed rate 0.5 mm s^−1^). The cutting process leads to smearing of material at the entrance and exit faces. Therefore the multilayer slices were further treated by focused ion beam (FIB) polishing, as illustrated in subfigure (*e*). Finally, two-dimensionally confining X-ray waveguides are obtained by gluing two polished waveguide slices on top of each other in a crossed geometry; see subfigure (*f*). Fig. 4(*a*) ▶ shows that the cutting process leads to smearing on the entrance and exit faces of the waveguide. Therefore the waveguide slices are polished by the FIB technique [FEI Nova Nanolab 600; Ga-ion source operating at 30 keV, ion current 5 nA, dwell time 300 ns, overlap (*x*-, *y*-direction) 50%, scan type ‘raster’ (Giannuzzi & Stevie, 2005 ▶)] to clean the Mo/C/Mo layer. Waveguide exit faces after FIB polishing with 35 nm- and 9 nm-thick guiding layers are exemplarily shown in Figs. 4(*b*) and 4(*c*) ▶ in 200k× and 300k× magnification, respectively.

The second technique used to realise an additional cap layer above the WG wafer is strainless electroless nickel plating (Enthone, ENfinity 4LF). Note that Ni offers higher absorption than In52Sn48 making the electroless nickel plating technique suitable for X-ray waveguides operating at energies up to 20 keV. In analogy to the bonding process, a 3 nm-thick Cr interlayer, which acts as an adhesive layer to an additional 120 nm-thick Ni layer, is deposited by electron beam evaporation on the WG cap layer. The Ni layer acts as a ‘metallic’ layer to optimize NiP precipitation on the WG wafer. The deposited NiP layer has a 2–4% P concentration and thus does not decrease the absorption properties significantly compared with a pure Ni layer. The maximum height of the NiP layer on top of the WG wafer is 300 µm. The waveguide is then cut into slices of length 1 mm and mechanically polished, using the transmission electron microscopy sample preparation technique, to the desired waveguide length. For the polishing, the WG slice is glued (Buehler, Cristalbond mounting wax 40-8150) onto a boro­silicate glass (Gebr. Rettberg GmbH) and successively polished with wet abrasive paper (Klingspor, kernel: PS11 P500C + P1000C; lubricant: water) and a diamond paste (Saint-Gobain GmbH, Winter diaplast SS D15-D1; lubricant: Winter diaplastol) on a dimpling disc (Buehler, Beta Grinder-Polisher) down to sub-5 µm face roughness. Finally, the entrance and exit sides of the waveguide slices are treated by FIB as in the case of the waveguide with bonded cap wafer.

## 4. Results: planar waveguides

**Figure 5:**
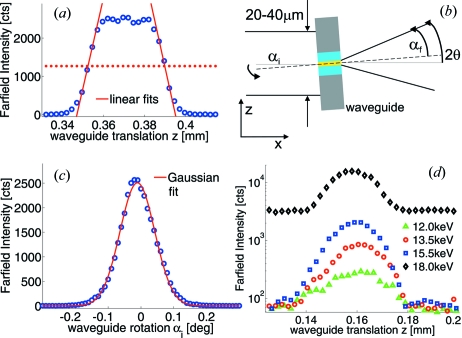
(*a*), (*c*) Integrated far-field intensity as a function of the waveguide translation yields a beam width of 37 µm and an angular acceptance of 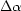 = 0.131° at 11.5 keV (35 nm C guiding layer, 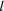 = 690 µm waveguide length). The InSn alloy acts as a beam-blocking material up to 15.5 keV at 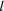 = 300 µm as no contributions of the primary beam are observed in the waveguide translation scans (*d*).

The experiments were performed at the BM20 bending-magnet beamline of the European Synchrotron Radiation Facility (ESRF, Grenoble, France) and at the P10 beamline of Petra III at HASYLAB (DESY, Hamburg, Germany). At BM20 we chose a wide energy range of 11.5–18.0 keV defined by a double Si(111) monochromator, placed in the middle of two conjugate Pt mirrors for higher harmonic rejection, and no pre-focusing optics to characterize the waveguides. The beam size was controlled by motorized entrance slits placed at a sub-15 cm distance to the waveguide and set to maximal 0.04 mm (vertical) × 2 mm (horizontal) with 1–3 × 10^7^ photons s^−1^ impinging on the waveguide in this configuration. Fig. 5(*a*) ▶ shows an example of the measured integrated far-field intensity with wide-opened detector slits as a function of the waveguide translation. The resulting width is a precise measure of the impinging beam, since the guiding layer *d* is vanishingly small. The width of the impinging beam in 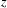 along with its intensity is needed to calculate the transmission efficiency of the waveguides. As expected for the alloy In52Sn48, the same scans at different energies for a 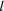 = 300 µm-long waveguide show no beam leakage at energies up to 15.5 keV (transmission of In and Ge: 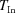 = 2.6 × 10^−4^, 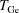 = 1.2 × 10^−6^) but contributions of the primary beam in the far-field intensity at 18.0 keV (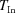 = 4.3 × 10^−3^, 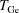 = 1.1 × 10^−4^).

**Figure 6:**
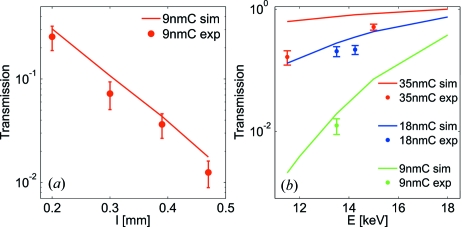
(*a*) The measured transmission 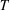 as a function of the waveguide length 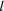 for a 9 nm WG at 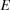 = 13.5 keV along with the simulated transmission. (*b*) The measured transmission 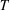 as a function of the energy 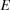 for waveguides of different guiding layer thicknesses at a waveguide length 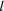 = (460 ± 10) µm.

The angular acceptance of the waveguides is in the range 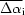 = 0.13–0.19° at energies between 11.5 and 15.0 keV (averaged over several measurements), determined by measuring the integrated far-field intensity as a function of the waveguide rotation; see Fig. 5(*c*) ▶. The results are summarized in Table 1 ▶. According to the theory, we would have expected a stronger decrease of the angular acceptance with higher energy and a slightly increased angular acceptance with larger guiding layer thicknesses, but such correlations are not clearly shown by the measurements. After careful alignment of the waveguide translation 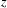, the angle of incidence 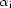 and the rotation around the optical axis χ (not shown), the transmission 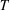 of the waveguides was measured (see Table 1 ▶). It depends on the waveguide length as well as on the thickness of the guiding layer C. It is defined as *T* = 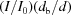, where 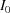 is the intensity impinging on the waveguide, 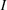 is the intensity exiting the waveguide, 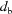 is the beam size of the incoming beam, and 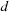 is the guiding layer thickness of the waveguide. The transmissions of the 35 nm and 18 nm C layer waveguide at 15.0 keV and 13.5 keV with waveguide lengths of 460 m and 300 m, respectively, are above 0.5 whereas the measured transmission of the 9 nm guiding layer waveguide is maximally 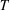 = 0.256 (at 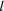 = 200 µm). The transmission 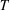 as a function of the waveguide length 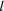 at 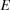 = 13.5 keV is shown in Fig. 6(*a*) ▶, indicating the significant transmission dependence of the 9 nm C guiding layer waveguide with 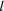. Likewise, Fig. 6(*b*) ▶ illustrates the strong dependence of the two-component waveguide transmission to the energy of the incoming synchrotron beam. The experimental results are in good agreement with the calculations of the FD simulations.

**Figure 7:**
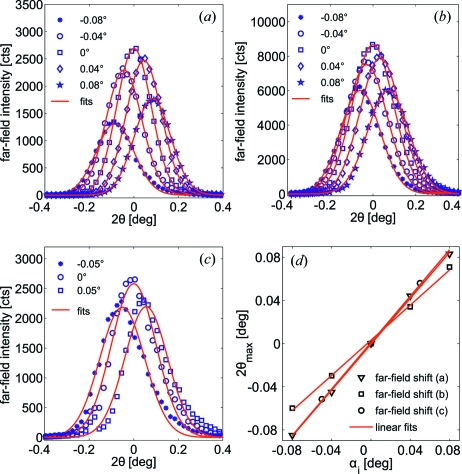
The far-field intensity distributions of the waveguides as a function of 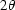 along with Gaussian fits [(*a*) 35 nm C, (*b*) 18 nm C, (*c*) 9 nm C]. The maxima of the far-field distributions are found approximately at 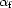 = 0, *i.e.* the slopes of 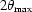 as a function of 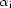 are near 1.

**Figure 8:**
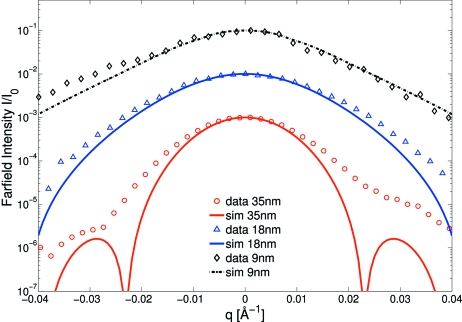
The normalized far-field intensity distributions of the waveguides as a function of the scattering vector 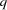 along with simulations (shifted for clarity). The far-fields are shifted for clarity. The FWHM obtained from Gaussian fits (not shown) are higher in the case of the mono-modal WG compared with the two-modal WG. The tails of the 9 nm WG are more pronounced than for the 18 nm WG far-field.

Fig. 7 ▶ shows the far-field intensity distributions of the waveguides of different guiding layer thickness as a function of 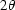 at varied angles of incidence 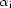. As expected for waveguide properties, by tilting the waveguide the maximum of the far-field intensity shifts by a corresponding angle. In principle, the FWHM of the far-field pattern increases with decreasing guiding layer thickness. Thus, the divergence of the waveguide exiting beam, *i.e.* the numerical aperture of a waveguide-based X-ray microscope, is enhanced. Fig. 8 ▶ shows the measured far-field distributions as a function of the wavelength-independent momentum transfer 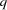 along with FD simulations. The FWHM 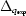 obtained by Gaussian fits are larger than expected from simulations. In the case of the 35 nm C guiding layer waveguide 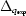 = 0.0185 Å^−1^ is 14% larger than the simulated value at 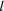 = 690 m. As described before, a 35 nm WG supports multiple modes, leading to a periodically alternating field distribution in the simulation. Depending on the exact length of the waveguide slice, varying exit fields and thus far-field patterns of different FWHM are obtained. This effect is less pronounced if the wavefield is more damped out in the cladding, *i.e.* at longer WG length and/or lower energy. At 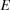 = 11.5 keV the simulated FWHM values are in the range 0.0155–0.0160 Å^−1^ for 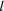 = 690 ± 15 m, and hence smaller than the measured value by at least 14%. Accordingly, the width of the near-field distribution must be lower than the simulated FWHM 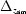 = 23.2 nm, *i.e.* 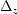 = 20.0 nm. The far-field width of the 18 nm C guiding layer waveguide 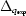 = 0.0257 Å^−1^ was measured 9% higher than calculated in the simulation 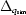 = 0.0233 Å^−1^ which leads to a near-field width of 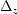 = 11.3 nm. The experimentally obtained divergence of the 9 nm C guiding layer waveguide differs by 2% from the expected value of the simulation with 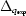 = 0.0254 Å^−1^ and 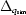 = 0.0248 Å^−1^. The corresponding width of the near-field distribution is 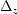 = 8.4 nm. In summary, the measured divergence of the WG beam increases substantially from a two-modal to a mono-modal WG, but the FWHM of the 9 nm WG is not larger than for the 18 nm WG. However, comparing the far-field distributions on a logarithmic scale, the measured algebraic tails decay much slower with smaller guiding layer thickness which may increase the effective numerical aperture and thus resolution in waveguide-based imaging.

## 5. Results: crossed waveguides

Two crossed waveguides (2DWG) were measured at the new endstation GINIX for coherent nano-focus imaging (Kalbfleisch *et al.*, 2010 ▶, 2011 ▶) installed at the P10 coherence beamline, Petra III (DESY, Hamburg, Germany). The endstation uses elliptically figured Pd-coated silicon and Pd-coated silica Kirkpatrick–Baez (KB) mirrors for vertical and horizontal focusing, respectively. The photon energy is defined by a fixed-exit double-crystal Si(111) monochromator, and can easily be changed without much re-alignment of the nano-focus. Some of the imaging experiments during the commissioning phase have been carried out at 7.9 keV, others at 13.8 keV, some at 15 keV. The parameters of the KB focus vary with energy, storage ring operation, and alignment status. During the 13.8 keV alignment, the focal spot size was measured to 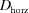 = 370 nm in the horizontal and 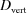 = 120 nm in the vertical direction, as measured by scanning the planar WG through the KB beam. The maximum integrated intensity in the focal spot of the KB beam was 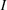 = 2.4 × 10^11^ photons s^−1^, as measured by a pixel detector (Pilatus 300K, Dectris). The waveguide was positioned in the focal spot of the KB system using a goniometer mounted upside down on a vibration-reduced extension arm with three miniaturized translations and two miniaturized rotations (Attocube Systems), along two directions, orthogonal to the optical axis 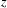. A more detailed description of the endstation can be found by Kalbfleisch *et al.* (2010 ▶). A noise-free single-photon-counting detector [Pilatus 300K, Dectris (Kraft *et al.*, 2009 ▶)] with a pixel size of 172 µm and an active area of 487 × 619 pixels was used to measure the far-field pattern of the WG at a distance of 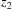 = 5.29 m.

**Figure 9:**
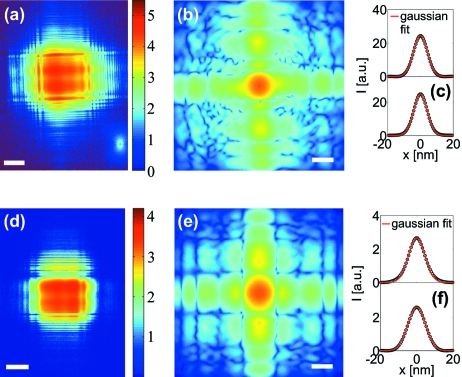
(*a*) Fraunhofer diffraction pattern of the 2DWG-1, pre-focused by KB mirrors, at 15.0 keV (logarithmic scale, scalebar 0.02 Å^−1^, 100 s dwell time). (*b*) WG near-field distribution in the effective confocal plane of the 2DWG-1, reconstructed using the ER algorithm (logarithmic scale, scalebar 20 nm). (*c*) Horizontal (top) and vertical (bottom) linescans (linear scale) of the reconstruction in (*b*) along with Gaussian fits yielding a width of 10.0 × 9.8 nm. (*d*) Far-field pattern of the 2DWG-2, measured at 13.8 keV (logarithmic scale, scalebar 0.02 Å^−1^, 1 s dwell time). (*e*) Reconstructed near-field of the 2DWG-2 (logarithmic scale, scalebar 20 nm). (*f*) Line profiles in the horizontal (top) and vertical (bottom) direction (linear scale), along with Gaussian fits yielding a width of 10.7 × 11.4 nm.

Fig. 9(*a*) ▶ shows the measured far-field pattern of a crossed waveguide system (2DWG-1) where the individual WG slices, denoted WG1-1 and WG2-1, have a guiding layer thickness of 35 nm each. The length of WG1-1 and WG2-1 are 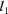 = 400 m and 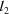 = 207 m, respectively, leading to a combined thickness of 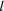 = 607 m. The incoming KB beam subsequently illuminated WG1 which was placed horizontally and WG2 which was placed vertically.

As described by Krüger *et al.* (2010 ▶), the 2DWG near-field was reconstructed using the iterative error-reduction (ER) algorithm. Fig. 9(*b*) ▶ shows the exit wavefield reconstruction after ten iterations of the ER algorithm. Note that the reconstructed near-field must be associated with an effective confocal plane of the 2DWG. Fig. 9(*c*) ▶ shows the line profile of the reconstruction in the horizontal (top) and vertical (bottom) direction along with Gaussian fits. The FWHM obtained from the fits are 10.0 nm and 9.8 nm in the horizontal and vertical direction, respectively. The high beam confinement is in agreement with the autocorrelation, which yields a FWHM of 18.3 × 17.8 nm. The respective FWHMs of the reconstruction are close to the values determined earlier for the same 2DWG, but with a different experimental set-up and at higher photon energy, reported by Krüger *et al.* (2010 ▶). The integrated photon flux exiting the 2DWG-1 was maximum at 2.0 × 10^7^ photons s^−1^.

Higher photon flux exiting a 2DWG can be reached by choosing a shorter waveguide length (adapted to the photon energy). We have performed the same measurements with a second crossed waveguide system, denoted as 2DWG-2, having a combined thickness of only 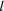 = 490 µm (WG1-2 vertically placed: 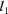 = 270 µm; WG2-2 horizontally placed: 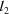 = 220 µm) at 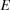 = 13.8 keV. Fig. 9(*d*) ▶ shows the measured far-field pattern of the 2DWG-2. The far-field pattern indicates similar characteristics as 2DWG-1. A maximum photon flux of 1.0 × 10^8^ photons s^−1^ exiting the 2DWG-2 was measured. In analogy to the reconstruction presented in Fig. 9(*b*) ▶, the near-field reconstruction shown in Fig. 9(*e*) ▶ exhibits a high beam confinement in the effective confocal plane of the 2DWG-2. Line scans with corresponding Gaussian fits yield a FWHM of 10.7 nm and 11.4 nm in the horizontal and vertical direction, respectively.

**Figure 10:**
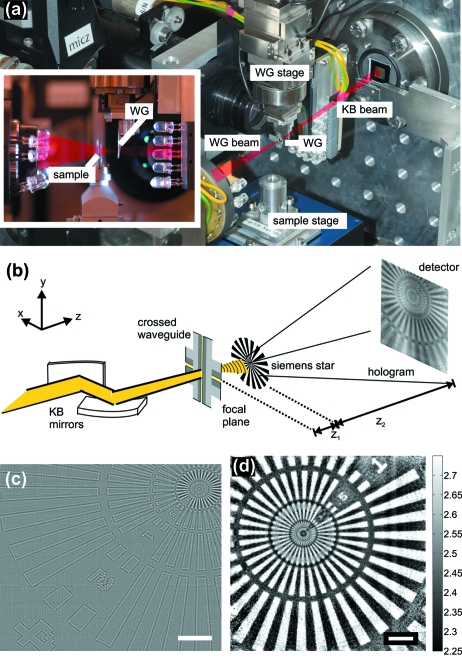
(*a*) Experimental set-up for coherent nano-focus imaging at the P10 beamline, Petra III. (*b*) Schematic of the experimental set-up for waveguide-based imaging using KB mirror pre-focusing. The sample is placed at a distance 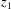 from the waveguide and the hologram is recorded at a distance 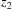 from the sample. (*c*) Holographic reconstruction of the Siemens star test structure after a combination of 7 × 6 scan points (scalebar 8 µm). (*d*) Phase reconstruction of a single image of the NTT pattern using a modified Gerchberg–Saxton algorithm (scalebar 4 µm).

Waveguides can be used as illumination source for propagation imaging in projection geometry, as demonstrated here for a test sample placed at a distance 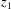 = 2.0 mm from the 2DWG-1 (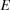 = 15 keV). The hologram is recorded at a distance 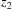 = 5.29 m from the sample using a single-photon-counting pixel detector (Pilatus, Dectris). Fig. 10(*a*) ▶ shows schematically the experimental set-up used at the P10 beamline (nano-focus endstation operated by University of Göttingen) for imaging of weakly scattering samples. The sample stage is equipped with a group of 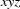 piezos (Physik Instrumente) on top of an air-bearing rotation (Micos). Additional 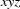 stages (Micos) below the rotation allow for distance variations of the sample to the WG. The distance between WG and sample is further controlled by two on-axis optical microscopes, one in front of the WG and one behind the sample.

Fig. 10(*c*) ▶ shows the holographic phase reconstruction of a Siemens star test pattern (NTT-AT, Japan; model ATN/XRESCO-50HC), recorded at the P10 beamline using the Pilatus pixel detector (Dectris). A mesh of 7 × 6 scan points was recorded with the sample shifted in the 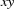-plane (exposure time 10 s each), *i.e.* a total number of 42 holograms.

Each hologram was reconstructed individually, and the resulting reconstructions were then stitched together. For holographic reconstruction the projection geometry used here was mapped onto parallel-beam propagation by a variable transformation based on the Fresnel scaling theorem. Given the distance 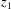 between the WG and the sample, parallel-beam reconstruction by Fresnel backpropagation of the recorded intensity can be applied using the effective defocus 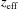 = 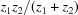 = 2.0 mm. At the same time the hologram is magnified corresponding to the geometrical projection by a factor of *M* = 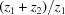 = 2646. Accordingly, given the 172 µm pixel size, the effective (de-magnified) pixel size in the sample plane is 65 nm. Corresponding to this sampling, the sector ring down to 100 nm lines and spaces is represented, but not the innermost sector ring down to the 50 nm lines and spacings. Holographic reconstruction is a robust one-step reconstruction scheme and the reconstruction is unique. However, the reconstructed phase distribution is adulterated by the so-called twin-image leading to artifacts, *i.e.* the reconstructed phase values are not quantitatively correct. For the present object and photon energy, a phase difference of 0.46 rad between the void areas and the Ta structure of the test pattern is expected. A pixel detector with smaller pixel size would thus improve the resolution at constant field of view, or allow for a larger field of view (as controlled by defocus distance) for constant resolution.

As another example, Fig. 10(*d*) ▶ shows an image (reconstructed phases), recorded at the ID22-NI undulator beamline of ESRF, using the same waveguide and test pattern, but in this case a pixel detector with 55 µm pixel size (Maxipix). The experimental set-up is described in detail by Krüger *et al.* (2010 ▶). At a defocus distance of 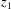 = 7 mm, the effective pixel size in the sample plane is 124.6  nm. Unfortunately, smaller 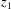 values were prohibited at this set-up by bulky positioning stages and sample mounts. The total photon flux impinging onto the sample was 7.6 × 10^7^ photons (17.5 keV, exposure time 1 s), providing a signal-to-noise ratio which is high enough for phase retrieval by an iterative algorithm. Compared with holographic reconstruction, iterative algorithms enable quantitative phase reconstruction without twin image artifacts. Here, we have used a modified Gerchberg–Saxton (GS) algorithm (Gerchberg & Saxton, 1972 ▶), enhanced by an additional reconstruction tool proposed by Marchesini *et al*. (2003 ▶) using a blurred version of the current estimate of the object under reconstruction. The blurring smoothes out noise and provides a form of regularization.

The blurring was carried out by convolving the reconstructed wavefield with a Gaussian of width σ at each iteration step. The width σ is set to 1 pixel (FWHM of 2.3548σ). The projection operator 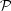 in the sample plane acts on the amplitude of the convolved estimate of the object 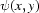,
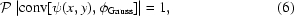
where conv denotes the convolution operator and 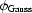 is a Gaussian of width σ. We denote this scheme as GS-Gaussian. The additional convolution reduces the spatial resolution of the reconstructed object owing to blurring. However, resolution can be recovered by subsequent GS iterations. Fig. 10(*c*) ▶ shows the phase reconstruction after 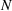 = 50 GS-Gaussian iteration steps followed by 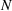 = 14 GS iteration steps. The two maxima of the phase histogram yield a relative phase shift of 0.38 rad, close to the expected phase shift of 0.4 rad.

The examples shown above show that the waveguide-based illumination system yields full-field phase-contrast hard X-ray images with adjustable magnification, resolution and field of view, at relatively low dose. Importantly, the waveguide acts as a coherence filter enhancing the image quality with respect to propagation imaging based on partially coherent illumination, or illumination systems with wavefront distortions. Rather than reconstructing both the wavefield and object, which is necessary for distorted phase fronts, a simple division by the empty beam yields very clean holograms. The disadvantage is a compromise in flux, and, as is always the case for high-magnification projection microscopy, a considerable sensitivity to mechanical vibrations. The theoretical resolution of the present waveguide system is in the range of 10 nm, corresponding to the beam confinement. This resolution range could not be reached or even tested in the present example, since the resolution was limited by pixel size as dictated by the defocus distance and detector pixel size. With improved instrumentation, in particular with high-resolution detectors, which have in the meantime been installed at the P10 nano-focus endstation, higher-resolution images are now in reach.

Finally, we comment on the astigmatism which is an intrinsic feature of the crossed WG system, leading in the present case to 200 µm offset between the vertical and horizontal source plane. At small 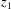 = 2 mm, as in the example shown in Fig. 10(*c*) ▶, this astigmatism leads to an optically visible ellipticity of about 10% in the reconstructed image. In future, we plan to remove this artifact by a simple generalization: the Fresnel propagators used in the reconstruction algorithms shall be adapted to the correct anisotropic propagation distance, each for the 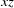 and 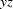 plane, respectively. However, this is beyond the scope of the present work which concentrates on waveguide fabrication and characterization, instead of imaging.

## 6. Conclusions

In summary, we have demonstrated optimized coherence and transmission properties of two-component planar X-ray waveguides. Novel fabrication techniques have been devised to reduce the waveguide length and enhanced the transmission, while maintaining coherence filtering and damping of radiation modes. We have studied the transmission as a function of photon energy and guiding layer thickness, both experimentally and by simulation. A maximum transmission of 0.26 has been measured for the 9 nm guiding layer waveguide at 13.5 keV which could be further enhanced by operating at higher energies. A crossed X-ray waveguide illumination system has been used for dose-efficient X-ray imaging at the nano-scale, as demonstrated at the new nano-focus endstation installed at the P10 beamline, Petra III. This highly coherent quasi-point source with two-dimensional beam confinement in the 10 nm range offers a homogeneous illumination wavefront for propagation imaging. The presented advances in waveguide design and parameters leads to a higher photon flux output at the waveguide exit, and thus enables three-dimensional imaging (tomography) of biological specimen, extending the work previously reported on the presented waveguide system (Giewekemeyer *et al.*, 2011 ▶).

## Figures and Tables

**Table 1:** Transmission and angular acceptance of planar Ge/Mo/C/Mo/Ge waveguides with guiding layer thicknesses 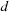 = 35 nm, 18 nm and 9 nm On the left: the experimentally obtained transmission 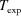 and the simulated transmission 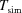 as a function of the photon energy 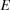 and the waveguide length 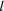. On the right: the mean angular acceptance 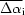 (FWHM) as a function of 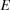. The 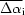 values are calculated from individual determinations of 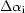 as obtained from angular acceptance measurements of waveguides of different lengths.

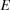 (keV)	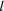 (µm)	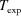	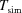	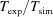 (%)	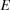 (keV)	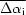 (°)
35 nm C layer						
11.50	460	0.165	0.627	26	11.50	0.131
11.50	690	0.140	0.847	29	13.50	0.164
15.00	460	0.512	0.847	60	15.0	0.129
15.00	690	0.379	0.714	53		

18 nm C layer						
12.50	300	0.177	0.413	43	13.50	0.190
12.50	480	0.083	0.197	42	18.00	0.153
13.00	300	0.287	0.462	62		
13.00	480	0.164	0.236	69		
13.50	300	0.315	0.513	61		
13.50	480	0.205	0.279	73		
14.00	300	0.404	0.565	72		
14.25	480	0.218	0.350	62		

9 nm C layer						
13.50	200	0.256	0.305	84	13.50	0.165
13.50	300	0.072	0.107	67	18.00	0.164
13.50	390	0.036	0.044	84		
13.50	470	0.013	0.018	71		
15.50	300	0.209	0.324	65		
